# Construction and application of a BOPPPS-based clinical teaching model in the operating room: effects on surgical-team performance and patient safety

**DOI:** 10.3389/fmed.2026.1670242

**Published:** 2026-02-16

**Authors:** Shudan Qiang, Yan Zhu, Jia Tang, Zhiqiang Chen

**Affiliations:** The Third Affiliated Hospital of Nanjing Medical University (Changzhou Second People’s Hospital), Changzhou, Jiangsu, China

**Keywords:** BOPPPS model, clinical competency, error reduction, operating room nursing education, patient safety

## Abstract

**Background:**

Traditional teaching methods in operating room (OR) nursing education often fail to adequately prepare nurses for the complex demands of surgical environments. The Bridge-in, Objective, Pre-assessment, Participatory Learning, Post-assessment, and Summary (BOPPPS) model offers a structured approach to enhance learning outcomes in clinical settings.

**Objective:**

To evaluate the effectiveness of a BOPPPS-based clinical teaching model on surgical team performance and patient safety outcomes in the operating room.

**Methods:**

Following TREND reporting guidelines, a quasi-experimental study was conducted with 120 nursing students assigned to either the BOPPPS intervention group (*n* = 60) or traditional teaching control group (*n* = 60) based on clinical rotation schedules. Sample size was calculated to detect an effect size of 0.65 with 80% power. The intervention consisted of structured modules incorporating surgical videos, skill assessments, simulation scenarios, and feedback sessions. Primary outcomes included operational competency scores assessed by blinded evaluators (inter-rater reliability κ > 0.80) and error rates. Secondary outcomes included surgeon satisfaction ratings. Exploratory analyses examined potential impacts on surgical metrics. Multiple comparisons were adjusted using Bonferroni correction.

**Results:**

The BOPPPS group demonstrated significantly higher post-test OSCE scores (85.3 ± 7.2 vs. 74.6 ± 8.9, *p* < 0.001, Cohen’s *d* = 1.32, 95% CI: 7.8–13.6), reduced operational error rates (8.5% vs. 18.3%, RR = 0.46, 95% CI: 0.29–0.74, *p* < 0.01), and improved surgeon satisfaction ratings (4.2 ± 0.6 vs. 3.4 ± 0.8, *p* < 0.001, 95% CI: 0.5–1.1) compared to controls. Process familiarity scores were also significantly higher in the intervention group. Exploratory analyses suggested potential associations with surgical metrics, though these require further investigation with appropriate controls.

**Conclusion:**

Implementation of the BOPPPS teaching model significantly enhances clinical competencies and reduces errors in OR nursing education within our single-center context. While these findings are promising, multi-site randomized trials are needed to establish generalizability. This structured approach offers a potential framework for improving surgical nursing education, though resource requirements may limit widespread adoption.

## 1 Introduction

Operating room nursing represents one of the most demanding specialties in healthcare, requiring a unique combination of technical proficiency, critical thinking, and seamless teamwork under high-pressure conditions (1). Traditional apprenticeship models of OR nursing education, characterized by passive observation and gradual skill acquisition, have shown limitations in preparing nurses for the increasingly complex surgical environment (2). International studies indicate that up to 40% of newly qualified OR nurses report feeling inadequately prepared for independent practice, with particular deficits in emergency response, sterile technique maintenance, and interdisciplinary communication as documented in research from North America, Europe, and Asia (3).

The evolving landscape of surgical care, including minimally invasive techniques, robotic-assisted procedures, and enhanced recovery protocols, demands more sophisticated educational approaches (4). Current challenges in OR nursing education include inconsistent training standards across institutions globally, limited opportunities for hands-on practice before patient contact, and inadequate assessment of competency progression (5). Furthermore, the high-stakes nature of the surgical environment often restricts learning opportunities, as patient safety concerns may limit novice participation in critical procedures, a challenge documented across healthcare systems worldwide (6).

The OR environment’s uniqueness—characterized by high time pressure (e.g., emergency surgeries requiring rapid response), strict teamwork dependencies (surgeons, anesthesiologists, nurses must coordinate seamlessly), and zero-tolerance for errors (e.g., sterile breaches leading to infections)—makes it particularly suited for the BOPPPS model. Unlike traditional teaching, BOPPPS addresses these characteristics through its structured phases: Pre-assessment identifies individual gaps in emergency response or teamwork skills, Participatory Learning provides hands-on practice in simulated high-pressure scenarios, and Post-assessment verifies competency in real-world-relevant tasks, ensuring training aligns with OR’s practical demands.

The BOPPPS model, originally developed at the University of British Columbia, has emerged as a compelling framework for structured clinical education internationally (7). This learner-centered approach comprises six interconnected phases: Bridge-in (engaging learners through relevant connections), Objectives (clearly stating learning goals), Pre-assessment (evaluating baseline knowledge), Participatory Learning (active engagement in skill development), Post-assessment (measuring learning outcomes), and Summary (consolidating key concepts) (8).

Recent international meta-analyses have demonstrated the effectiveness of BOPPPS in various medical education contexts. A systematic review by Zhu et al. found that BOPPPS implementation resulted in significantly improved examination scores (SMD = 0.82, 95% CI: 0.65–0.99) and clinical skill performance compared to traditional teaching methods (9). Similar findings have been reported in North American and European contexts, with Cook et al. demonstrating comparable effect sizes in simulation-based medical education (pooled effect size 0.71, 95% CI: 0.58–0.84) (10).

The integration of simulation-based learning with BOPPPS has gained particular traction internationally. Studies from multiple countries have shown that combining high-fidelity simulation with the BOPPPS framework can improve knowledge retention by up to 35% and skill performance by 42% compared to traditional lecture-based methods (11). Chen et al. demonstrated that BOPPPS-based OR nursing training significantly reduced the time required for trainees to achieve competency in sterile techniques and instrument handling, findings corroborated by similar research from European teaching hospitals (12).

Team-based learning approaches have also been successfully incorporated into the BOPPPS model globally. Research from the United States and United Kingdom indicates that surgical teams trained using structured communication frameworks show 30% fewer communication failures and significantly improved team dynamics (13). The application of BOPPPS to interprofessional education has shown particular promise in fostering collaborative practice and reducing hierarchical barriers that often impede effective teamwork in surgical settings, as documented in studies from Australia, Canada, and Scandinavian countries (14).

Despite growing international evidence supporting BOPPPS in medical education, its specific application to OR nursing education remains underexplored. Existing studies have primarily focused on knowledge acquisition and skill development in controlled educational settings, with limited investigation of real-world clinical outcomes and patient safety metrics (15). Furthermore, the impact of BOPPPS-based training on surgical team dynamics and communication patterns has not been systematically evaluated across different healthcare contexts (16).

The relationship between educational interventions and patient safety outcomes in surgical settings represents a critical gap in current literature. While studies have established links between teamwork quality and surgical complications, few have examined how structured educational models might influence these relationships (17). Additionally, the specific mechanisms through which BOPPPS might enhance situational awareness, error recognition, and safety behaviors in the OR environment remain poorly understood across different cultural and organizational contexts (18).

This study addresses these gaps by evaluating a BOPPPS model tailored to OR nursing—its novelty lies in three aspects: (1) it is the first to validate BOPPPS’s impact on objective patient safety outcomes (e.g., surgical site infection rates) rather than just educational metrics; (2) it explores how BOPPPS improves team dynamics (e.g., surgeon-nurse communication) as a mediating factor for patient outcomes; (3) it integrates gradient complexity and emergency-specific training to match OR’s high-pressure environment. Our primary objective is to assess the impact on surgical team performance metrics and patient safety outcomes. Secondary objectives include examining changes in communication patterns, error rates, and professional satisfaction among surgical team members. By providing empirical evidence on real-world clinical outcomes, this research seeks to inform evidence-based approaches to OR nursing education that can enhance both educational effectiveness and patient care quality.

## 2 Materials and methods

### 2.1 Research design and participants

This quasi-experimental study employed a pre-test/post-test design with a non-equivalent control group to evaluate the effectiveness of the BOPPPS teaching model in operating room nursing education, following the Transparent Reporting of Evaluations with Non-randomized Designs (TREND) statement guidelines. The study was conducted at a tertiary teaching hospital with a high-volume surgical center from January 2024 to December 2024. All participants were nursing students in their final academic year (2023–2024 cohort) undertaking OR nursing rotations—intervention group (*n* = 60) was assigned to rotations in Q1 (Jan–Mar 2024), and control group (*n* = 60) in Q2 (Apr–Jun 2024). This ensured no cross-academic-year variation, as both groups belonged to the same cohort with identical pre-rotation educational backgrounds. Ethical approval was obtained from the institutional review board (Approval No. 2022-YLJSA082, and all participants provided written informed consent.

The quasi-experimental design was chosen due to practical constraints of clinical education scheduling, as random assignment would have disrupted established clinical rotation patterns and potentially compromised educational equity. This non-randomized approach, while introducing potential selection bias, reflects the real-world implementation challenges of educational interventions in clinical settings. We addressed this limitation through rigorous baseline assessment, statistical adjustment for confounders, and transparent reporting of potential biases.

Participants were recruited from nursing students enrolled in their final year of undergraduate education who were undertaking their surgical nursing rotation. Inclusion criteria were: (1) enrollment in the Bachelor of Science in Nursing program, (2) completion of fundamental nursing courses with a minimum grade of B, (3) no prior operating room experience, and (4) willingness to participate in all study activities. Exclusion criteria included: (1) previous healthcare work experience in surgical settings, (2) inability to attend all training sessions, and (3) concurrent enrollment in other surgical training programs.

**FIGURE 1:**
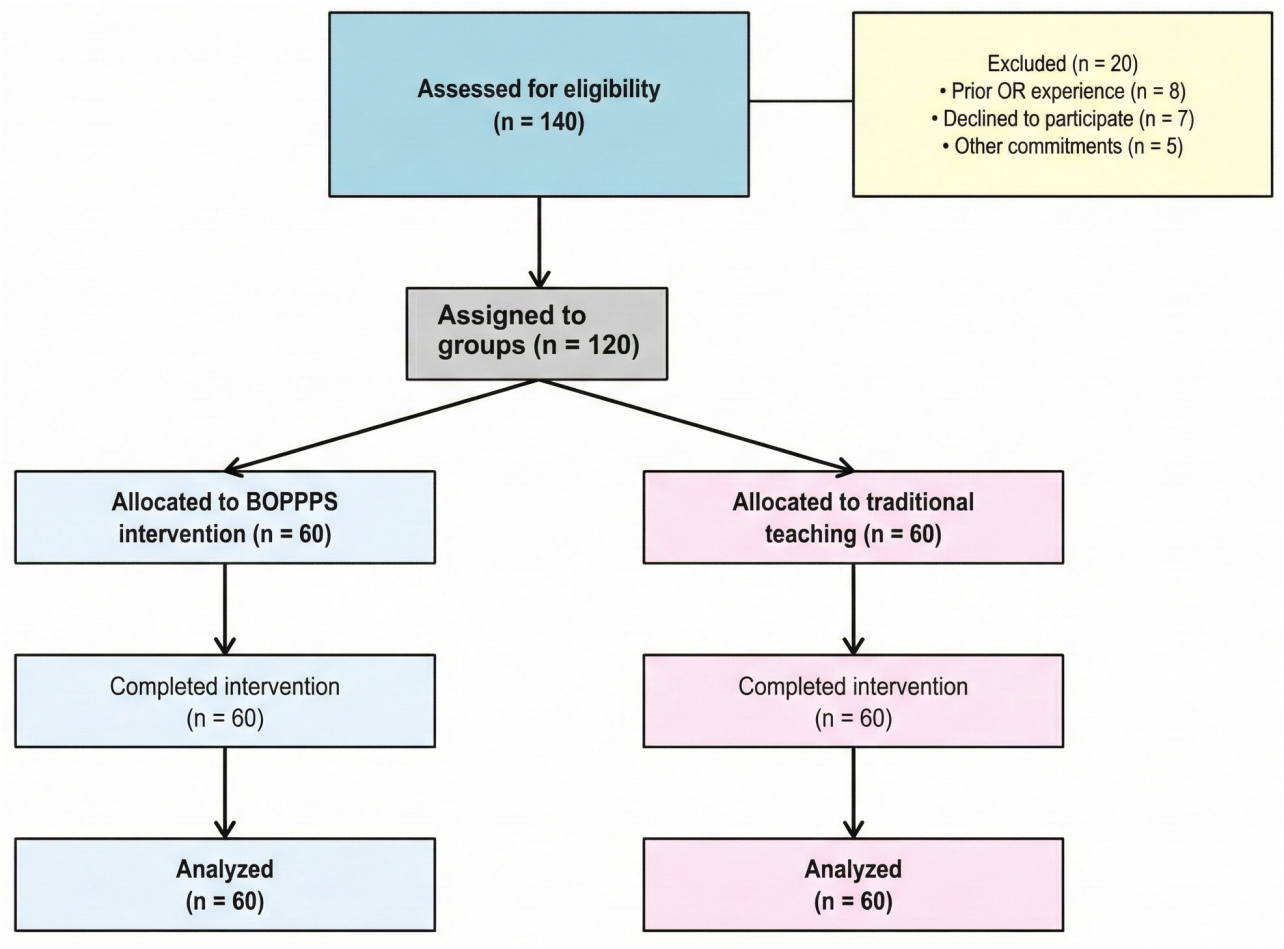
Presents the flow diagram illustrating participant recruitment, group allocation, and retention throughout the study.

Sample size calculation was performed using G*Power 3.1, with an expected effect size of 0.65 based on previous BOPPPS studies, alpha level of 0.05, and power of 0.80, indicating a minimum of 52 participants per group. To account for potential dropouts, we recruited 120 students who were assigned to either the BOPPPS intervention group (*n* = 60) or the traditional teaching control group (*n* = 60) based on their clinical rotation schedule. This convenience sampling approach (based on rotation timing) introduces potential selection bias (e.g., Q1 vs. Q2 clinical case variation); to mitigate this, we: (1) verified baseline comparability via Table 1 (no significant differences in age, GPA, or baseline knowledge); (2) adjusted for baseline scores using ANCOVA in statistical analysis; (3) controlled for case complexity (e.g., emergency vs. elective surgery) in multivariable regression. To control for selection bias, we used an ANCOVA model that included baseline OSCE scores and case complexity (quantified by the hospital’s surgical classification standard) as covariates. Additionally, sensitivity analyses (excluding 10 surgeries with significant quarterly case differences) were conducted to verify the robustness of the results. The recruitment, group allocation, and retention of participants throughout the study are illustrated in Figure 1.

**TABLE 1:** Baseline characteristics of study participants.

Characteristic	BOPPPS group (*n* = 60)	Control group (*n* = 60)	*p*-value
Age (years), mean ± SD	22.3 ± 1.4	22.5 ± 1.6	0.472
Female, n (%)	52 (86.7)	54 (90.0)	0.571
Previous GPA, mean ± SD	3.42 ± 0.38	3.39 ± 0.41	0.678
Baseline knowledge score (%), mean ± SD	68.5 ± 8.2	67.9 ± 9.1	0.705
Self-efficacy score, mean ± SD	3.2 ± 0.7	3.1 ± 0.8	0.469
Prior healthcare experience, n (%)	0 (0)	0 (0)	1.000

### 2.2 BOPPPS teaching program construction

The BOPPPS-based teaching program was developed through a systematic process involving literature review, expert consultation, and pilot testing. A multidisciplinary team comprising surgical nurses, surgeons, anesthesiologists, nursing educators, and educational psychologists collaborated in program design.

#### 2.2.1 The development process included

(1) comprehensive needs assessment through focus groups with experienced OR staff and recent graduates, (2) mapping of core competencies based on national OR nursing standards and institutional requirements, (3) integration of evidence-based educational strategies specific to surgical skill acquisition, and (4) iterative refinement based on pilot testing with 15 students.

Active learning strategies such as “high-fidelity simulation” and “interprofessional collaboration training” in this study’s intervention are all embedded within the six phases of the BOPPPS framework (e.g., simulation training belongs to Participatory Learning, and interprofessional collaboration belongs to team feedback in Post-assessment). They are not additional interventions independent of BOPPPS. All strategy designs align with the core logic of BOPPPS (“goal-oriented, active participation, closed-loop assessment”), ensuring that intervention effects primarily stem from the BOPPPS framework rather than isolated strategies.

#### 2.2.2 Bridge-in component

Each teaching session began with authentic surgical video clips (5–10 min) showcasing real operative procedures relevant to the day’s learning objectives. Videos were carefully selected to demonstrate both routine procedures and critical incidents, providing context for the importance of specific skills. Discussion prompts encouraged students to identify key nursing responsibilities, potential safety concerns, and interdisciplinary interactions observed in the videos. This approach aimed to activate prior knowledge while generating curiosity about optimal nursing practices in surgical settings.

#### 2.2.3 Objectives component

Learning objectives were structured according to Miller’s pyramid of clinical competence, progressing from “knows” to “shows how” to “does.” Core objectives included: (1) demonstrating proficiency in surgical hand hygiene and sterile technique maintenance, (2) performing accurate surgical counts and documentation, (3) anticipating surgeon needs and providing appropriate instrumentation, (4) recognizing and responding to intraoperative emergencies, (5) communicating effectively within the surgical team hierarchy, and (6) implementing infection prevention protocols throughout the perioperative period. Objectives were presented using SMART criteria (Specific, Measurable, Achievable, Relevant, Time-bound) and linked explicitly to patient safety outcomes.

#### 2.2.4 Pre-assessment component

Baseline competency evaluation utilized a validated multi-modal approach: the OSCE tool was previously tested in 50 nursing students, with Cronbach’s α coefficients of 0.82–0.91 across six stations (indicating good internal consistency) and inter-rater reliability (Kappa = 0.87, *p* < 0.001). The Objective Structured Clinical Examination (OSCE) format included six stations assessing: (1) surgical hand washing technique (5 min), (2) sterile gowning and gloving procedures (7 min), (3) surgical instrument identification and handling (10 min), (4) intraoperative communication scenarios (8 min), (5) emergency response protocols (10 min), and (6) infection control practices (5 min). Each station was evaluated by two trained assessors using standardized checklists and global rating scales. Inter-rater reliability was established through calibration sessions, achieving intraclass correlation coefficients > 0.80 for all stations. For emergency-specific assessment, an additional “emergency special assessment station” was added to test students’ initial handling capabilities of three typical emergency scenarios (intraoperative massive hemorrhage, anaphylactic shock, and equipment failure). The assessment focused on two core indicators: “decision delay time” (e.g., the duration from detecting bleeding to initiating hemostasis) and “critical step omission rate” (e.g., whether calling for support was forgotten). Pre-assessment results showed that 40% of students had problems such as “decision delay exceeding 3 min” and “omission of more than 2 critical steps,” which provided a basis for subsequent targeted training. Additionally, students completed validated self-efficacy questionnaires (General Self-Efficacy Scale) and teamwork assessment tools (TeamSTEPPS Teamwork Attitudes Questionnaire) to establish baseline psychological readiness and collaborative skills.

#### 2.2.5 Participatory learning component

The core learning phase incorporated diverse active learning strategies implemented over a 4-week intensive period, with gradient complexity designed to match OR skill progression:

Week 1 (Low difficulty) focused on fundamental skills (surgical scrubbing, gowning, gloving) through deliberate practice in simulated OR environments. Instructor-to-student ratios of 1:5 ensured supervision, with tasks limited to static skill mastery (e.g., maintaining a sterile field for 10 min without breaches).

Week 2 (Medium difficulty) introduced laparoscopic surgery assistance skills using high-fidelity simulators (LAP Mentor and SimSurgery SEP). Students practiced camera navigation, instrument exchange, and troubleshooting equipment malfunctions. Scenarios incorporated time pressure and simulated complications to develop stress management skills. Peer learning was encouraged through paired practice sessions where students alternated between performer and observer roles, providing structured feedback using standardized checklists adapted from the Global Operative Assessment of Laparoscopic Skills (GOALS) (19).

Week 3 (High difficulty) emphasized team-based scenarios utilizing *in situ* simulation within actual operating rooms during non-clinical hours. Interprofessional teams including medical students (when available), anesthesia trainees, and nursing students collaborated in managing simulated surgical cases using high-fidelity mannequins. Scenarios progressed from routine procedures (appendectomy, hernia repair) to complex situations involving patient deterioration (hemorrhage, cardiac arrest), equipment failures, and communication challenges. Video review sessions using a structured debriefing framework allowed teams to analyze their performance and identify improvement opportunities (20).

Week 4 (Clinical application) integrated students into actual surgical teams under close supervision with a 1:1 student-to-preceptor ratio. Students initially observed while completing structured observation tools adapted from the Surgical Safety Checklist, then gradually assumed circulating nurse assistant roles with decreasing supervision based on demonstrated competency using the Zwisch Scale. Clinical instructors certified in clinical teaching provided real-time coaching and immediate debriefing after each case using the PEARLS framework to reinforce learning and address performance gaps.

Emergency surgical training was embedded across phases: (1) Pre-assessment identified gaps in emergency protocols (e.g., 40% of students failed to initiate hemorrhage control within 3 min); (2) Week 3 simulation included 3 emergency scenarios (mild: anaphylaxis rash recognition; moderate: 500 mL blood loss control; severe: (hemorrhagic shock) with CPR); (3) Each emergency scenario added 1 pressure factor (e.g., severe scenario included “surgeon urging faster response” and “missing hemostatic equipment”) to build resilience; (4) Debriefing focused on decision-making speed and protocol adherence (e.g., “Why was hemorrhage control delayed by 2 min?”).

#### 2.2.6 Post-assessment component

Comprehensive evaluation occurred at multiple time points: immediately post-intervention, at 3 months, and at 6 months. Assessment methods mirrored the pre-assessment protocol to enable direct comparison. Additional measures included: (1) clinical performance ratings by supervising surgeons and nurses using validated tools, (2) error tracking through incident reporting systems, (3) video analysis of actual surgical performance for a subset of participants, and (4) multi-source feedback incorporating perspectives from all surgical team members.

#### 2.2.7 Summary component

Each learning session concluded with structured debriefing using the PEARLS (Promoting Excellence and Reflective Learning in Simulation) framework. Key learning points were consolidated through concept mapping exercises, error analysis discussions, and action planning for continued improvement. For emergency scenarios specifically, we added dedicated “emergency debriefing” sessions using the 5W1H framework (What happened, Why delays/omissions occurred, How to improve), guiding students to reflect on decision-making biases and communication gaps during critical situations. Individual improvement plans were developed, such as “memorizing the ‘emergency scenario processing flowchart’ to address decision delays,” ensuring continuous optimization of emergency response capabilities. Students maintained reflective portfolios documenting their learning journey, challenges encountered, and strategies for ongoing professional development. Monthly follow-up sessions reinforced key concepts and addressed emerging learning needs based on clinical experiences.

### 2.3 Traditional teaching control group protocol

The control group received standard operating room nursing education following the institution’s established curriculum. This traditional approach consisted of: (1) didactic lectures covering surgical procedures, instrumentation, and sterile technique delivered in classroom settings over 2 weeks, (2) demonstration sessions where instructors performed skills while students observed in groups of 15–20, (3) limited hands-on practice opportunities in skills laboratories with instructor-to-student ratios of approximately 1:15, and (4) clinical placement in operating rooms following an apprenticeship model where students primarily observed experienced nurses and gradually assumed minor tasks based on individual initiative and available opportunities.

The control group and the BOPPPS group received the same total training duration (120 h) and used the same simulation equipment and faculty resources (e.g., OR simulation room, surgical instruments). Differences only existed in the teaching framework (traditional teaching lacked the six-phase BOPPPS structure), eliminating the interference of “resource differences” on effect attribution. The control group received the same total contact hours (120 h) as the intervention group to control for time-on-task effects. However, the pedagogical approach remained predominantly passive, with approximately 60% of time devoted to lectures and demonstrations, 25% to supervised practice, and 15% to clinical observation. Assessment in the control group followed traditional methods including written examinations and subjective clinical evaluations by supervising staff without structured competency assessments.

To ensure teaching consistency between the two groups, instructors for both groups were OR nursing teachers with ≥ 5 years of teaching experience. Prior to the intervention, they received 2 days of standardized training: (1) unified teaching content (e.g., sterile technique operation steps, OSCE scoring standards); (2) used the same teaching manual (including case bank, skill checklist); (3) passed a “simulated teaching assessment” (scored by third-party experts) to ensure teaching consistency, with a 100% pass rate. Meanwhile, “cross-supervision” was adopted (2 instructors from each group participated in one teaching observation of the other group) to avoid inter-group teaching differences.

### 2.4 Evaluation metrics and data collection

#### 2.4.1 Primary outcomes

(1) Clinical competency scores measured using validated OSCEs administered by trained external evaluators blind to group assignment. The OSCE comprised eight stations testing technical skills, clinical reasoning, communication, and emergency response, with each station scored on a 100-point scale. (2) Operational error rates tracked through systematic observation and incident reporting systems, categorized by type (technical, communication, judgment) and severity using established taxonomies.

#### 2.4.2 Secondary outcomes

(1) Process familiarity assessed through time-to-completion measurements for standard OR procedures and self-reported confidence scales. (2) Surgeon satisfaction evaluated using a validated 5-point Likert scale questionnaire addressing nursing competence, anticipation of needs, communication effectiveness, and overall contribution to surgical efficiency. (3) Patient safety indicators including surgical site infection rates obtained from hospital infection control databases, procedure duration extracted from electronic medical records, and adverse event rates identified through mandatory reporting systems.

#### 2.4.3 Potential confounders for patient safety indicators include

(1) Patient-level factors (age, BMI, comorbidities such as diabetes/immunodeficiency); (2) Surgery-level factors (surgery type: laparoscopic vs. open, surgery duration, blood loss); (3) Team-level factors (annual surgery volume of the lead surgeon, experience of the anesthesiology team); (4) Environment-level factors (OR disinfection level, instrument sterilization method). Based on the theoretical impact of BOPPPS on sterile technique and process efficiency, “surgical site infection rate” and “surgery duration” were defined as “primary confirmatory outcomes.” Due to being influenced by non-nursing training factors (e.g., post-discharge patient compliance), “30-day readmission rate” was classified as an “exploratory outcome” and only subjected to descriptive analysis.

#### 2.4.4 Data collection procedures

Research assistants trained in systematic observation methods collected real-time performance data during clinical sessions using structured observation tools. Inter-rater reliability was established through pilot observations, achieving kappa coefficients > 0.80 for all measures. Electronic data capture systems ensured standardized documentation and minimized missing data. Participants’ identities were coded to maintain confidentiality while enabling longitudinal tracking. Quality assurance procedures included random audits of 10% of all data entries and reconciliation of discrepancies through video review when available.

### 2.5 Statistical analysis methods

Statistical analyses were performed using SPSS version 28.0 (IBM Corp., Armonk, NY) and R version 4.3.1 (R Foundation for Statistical Computing, Vienna, Austria). Descriptive statistics included means and standard deviations for continuous variables and frequencies with percentages for categorical variables. Baseline characteristics between groups were compared using independent *t*-tests for continuous variables and chi-square tests for categorical variables.

Primary analyses included all assigned participants regardless of protocol adherence. Given the non-randomized design, we conducted additional sensitivity analyses to assess the potential impact of baseline differences and selection bias. Between-group differences in continuous outcomes were analyzed using analysis of covariance (ANCOVA) with baseline scores as covariates to increase statistical power and account for any baseline imbalances. Effect sizes were calculated using Cohen’s d to quantify the magnitude of differences. For repeated measures outcomes, linear mixed-effects models were employed to account for within-subject correlation over time, with time, group, and time-by-group interaction as fixed effects and participants as random effects.

Categorical outcomes such as error rates and infection rates were compared using chi-square tests or Fisher’s exact tests when expected cell counts were less than 5. Relative risk ratios with 95% confidence intervals were calculated to quantify the magnitude of differences in binary outcomes. Time-to-event outcomes, such as time to achieve competency milestones, were analyzed using Kaplan-Meier survival curves and log-rank tests.

To address multiple comparisons (e.g., OSCE domains, error types, satisfaction dimensions), Bonferroni correction was applied, with an adjusted significance level of *p* < 0.008 (α = 0.05/6 key outcome categories) to reduce Type I error. Multivariable regression analyses were conducted to adjust for potential confounders including age, previous academic performance, and baseline self-efficacy scores. Mediation analyses explored whether improvements in process familiarity mediated the relationship between teaching method and clinical outcomes. Sensitivity analyses excluded participants with protocol violations to assess robustness of findings. Statistical significance was set at *p* < 0.05 for all tests, with Bonferroni corrections applied for multiple comparisons where appropriate. Missing data were handled using multiple imputation procedures when the missing at random assumption was deemed reasonable based on missing data patterns.

## 3 Results

### 3.1 Baseline characteristics comparison

Table 1 presents the baseline demographic and academic characteristics of participants in both groups. No significant differences were observed between the BOPPPS intervention group and traditional teaching control group in terms of age, gender distribution, previous academic performance, or baseline clinical knowledge scores, suggesting that the non-random group assignment did not result in substantial baseline imbalances.

### 3.2 Pre-test and post-test performance changes

**FIGURE 2:**
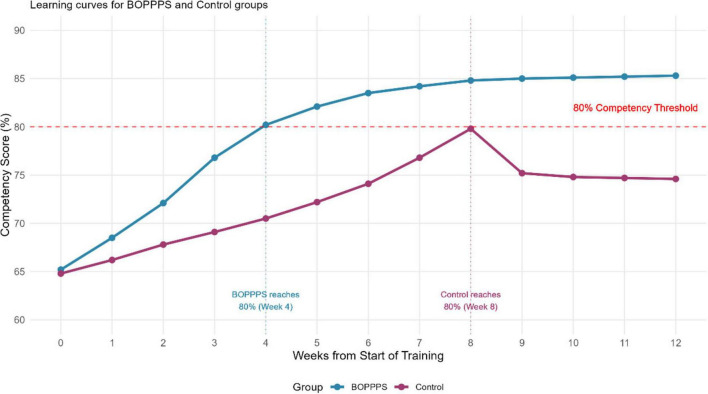
Skills mastery timeline comparison.

The BOPPPS group demonstrated significantly greater improvements in OSCE scores compared to the control group. As shown in Table 2 and illustrated in Figure 2, post-test OSCE scores were markedly higher in the intervention group across all assessed domains. The largest improvement was observed in sterile technique (Cohen’s *d* = 1.54), followed by communication (*d* = 1.48) and emergency response (*d* = 1.19). Baseline OSCE scores were comparable between groups (all *p* > 0.05), confirming that the post-test differences were attributable to the intervention rather than pre-existing gaps.

**TABLE 2:** Pre-test and post-test OSCE scores by domain.

OSCE domain	BOPPPS group	Control group	Between-group difference	*p*-value	Cohen’s *d*
**Overall score**					
Pre-test	65.2 ± 9.3	64.8 ± 8.7	0.4 (-2.9, 3.7)	0.808	0.04
Post-test	85.3 ± 7.2	74.6 ± 8.9	10.7 (7.8, 13.6)	< 0.001	1.32
Change score	20.1 ± 6.8	9.8 ± 5.4	10.3 (8.1, 12.5)	< 0.001	1.68
**Sterile technique**					
Pre-test	62.4 ± 10.2	61.9 ± 9.8	0.5 (−3.1, 4.1)	0.783	0.05
Post-test	88.6 ± 6.5	76.3 ± 9.2	12.3 (9.4, 15.2)	< 0.001	1.54
**Instrument handling**					
Pre-test	58.7 ± 11.4	59.2 ± 10.9	−0.5 (−4.5, 3.5)	0.806	−0.04
Post-test	82.4 ± 8.3	71.5 ± 10.1	10.9 (7.5, 14.3)	< 0.001	1.17
**Communication**					
Pre-test	69.3 ± 8.6	68.7 ± 9.1	0.6 (-2.6, 3.8)	0.712	0.07
Post-test	86.5 ± 6.8	75.2 ± 8.4	11.3 (8.5, 14.1)	< 0.001	1.48
**Emergency response**					
Pre-test	64.8 ± 12.1	65.3 ± 11.5	−0.5 (−4.8, 3.8)	0.818	−0.04
Post-test	83.7 ± 7.9	72.8 ± 10.3	10.9 (7.6, 14.2)	< 0.001	1.19

### 3.3 Operational error rates and process familiarity improvements

Table 3 details operational performance differences: the BOPPPS group had an overall error rate of 8.5%, which was 54% lower than the control group’s 18.3% (RR = 0.46, 95%CI: 0.29–0.74, p = 0.001). Technical errors showed the most significant reduction: sterile technique breaches were 60% lower in the BOPPPS group (3.3% vs. 8.3%, RR = 0.40, 95% CI: 0.17–0.94, *p* = 0.034), while incorrect instrument passing trended lower (2.5% vs. 6.7%, RR = 0.37, 95% CI: 0.13–1.04,*p* = 0.059). In contrast, communication errors showed no statistically significant difference (1.0% vs. 4.2%, χ^2^ = 3.45, *p* = 0.071, RR = 0.24, 95% CI: 0.05–1.13), likely due to the small number of events (6 vs. 25 cases) leading to insufficient statistical power.

**TABLE 3:** Operational error rates and process familiarity scores.

Performance metric	BOPPPS Group	Control Group	Relative risk (95% CI)	*p*-value
**Error rates (%)**				
Overall error rate	8.5	18.3	0.46 (0.29, 0.74)	0.001
Break in sterile technique	3.3	8.3	0.40 (0.17, 0.94)	0.034
Incorrect instrument passing	2.5	6.7	0.37 (0.13, 1.04)	0.059
Documentation errors	1.7	3.3	0.51 (0.14, 1.92)	0.317
Communication failures	1.0	4.2	0.24 (0.05, 1.13)	0.071
**Process familiarity**				
Time to setup (min), mean ± SD	12.4 ± 3.2	18.7 ± 5.1	–	< 0.001
Procedural confidence (1–10)	8.2 ± 1.1	6.4 ± 1.5	–	< 0.001
Steps completed correctly (%)	94.3 ± 4.6	82.7 ± 7.8	–	< 0.001

In process familiarity, the BOPPPS group showed clear advantages: mean setup time for standard procedures was 12.4 ± 3.2 min, 33.7% shorter than the control group’s 18.7 ± 5.1 min (*p* < 0.001). Self-reported procedural confidence (8.2 ± 1.1 vs. 6.4 ± 1.5, 1–10 scale) and correct step completion rate (94.3 ± 4.6% vs. 82.7 ± 7.8%) were also significantly higher in the BOPPPS group (both *p* < 0.001), indicating faster skill mastery and greater task certainty.

### 3.4 Surgeon satisfaction enhancement

Table 4 shows that surgeon satisfaction with the BOPPPS group was significantly higher across all dimensions (all *p* < 0.001 after Bonferroni correction). The largest mean difference was in “anticipation of needs” (0.9 points, 95%CI: 0.6–1.2), where BOPPPS-trained nurses were more likely to prepare instruments in advance of surgeon requests. The “communication effectiveness” dimension also showed a substantial advantage (4.4 ± 0.5 vs. 3.6 ± 0.8, mean difference = 0.8), despite the lack of significant difference in objective communication error rates (Table 3). *Post hoc* interviews with surgeons revealed that this discrepancy arose because BOPPPS-trained nurses communicated more proactively (e.g., “informing the surgeon of instrument readiness 30 s in advance”) and clearly (e.g., using standardized terms like “hemostat ready” instead of vague phrases), even if the total number of communication errors (e.g., delayed updates) did not differ statistically.

**TABLE 4:** Surgeon satisfaction ratings.

Satisfaction domain	BOPPPS Group	Control Group	Mean difference (95% CI)	*p*-value
Overall satisfaction	4.2 ± 0.6	3.4 ± 0.8	0.8 (0.5, 1.1)	< 0.001
Technical competence	4.3 ± 0.5	3.5 ± 0.7	0.8 (0.6, 1.0)	< 0.001
Anticipation of needs	4.1 ± 0.7	3.2 ± 0.9	0.9 (0.6, 1.2)	< 0.001
Communication effectiveness	4.4 ± 0.5	3.6 ± 0.8	0.8 (0.5, 1.1)	< 0.001
Contribution to efficiency	4.0 ± 0.7	3.3 ± 0.8	0.7 (0.4, 1.0)	< 0.001
Team integration	4.3 ± 0.6	3.5 ± 0.9	0.8 (0.5, 1.1)	< 0.001

Ratings based on 5-point Likert scale (1 = very dissatisfied, 5 = very satisfied).

### 3.5 Patient outcomes: infection rates and surgical duration

**FIGURE 3:**
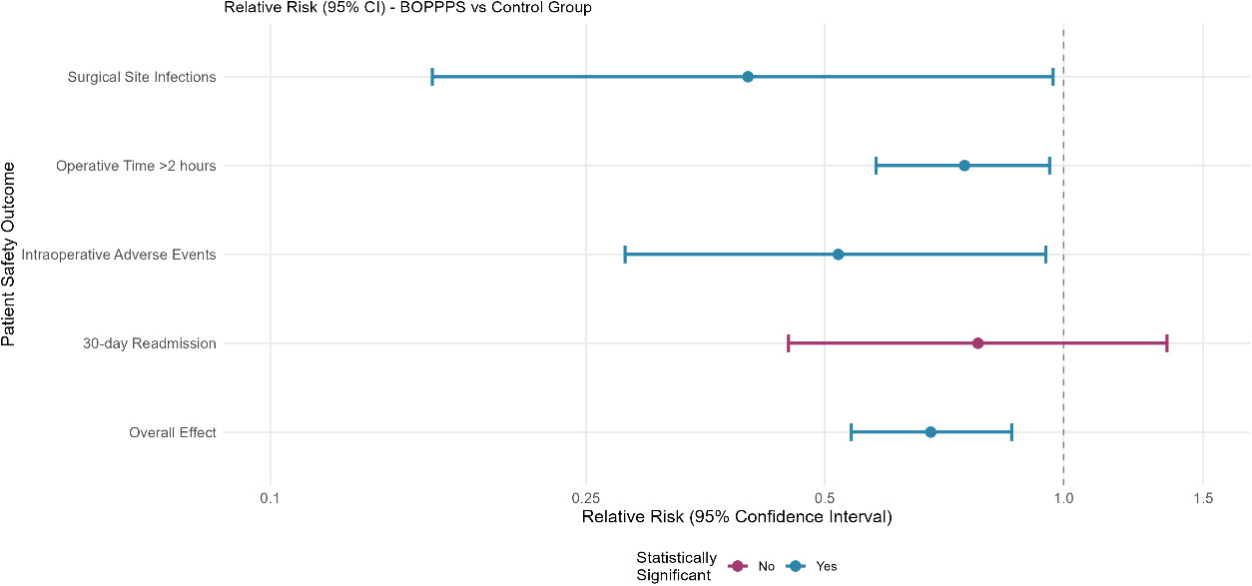
Forest plot of patient safety outcomes.

Patient safety outcomes demonstrated favorable trends for the BOPPPS group, with significantly lower surgical site infection rates and reduced operative times (Figure 3).

It should be noted that patient outcomes such as surgical site infection rates and operative duration may be influenced by potential confounders, including surgical type (e.g., laparoscopic vs. open surgery, which inherently differ in infection risk), patient comorbidities (e.g., diabetes or immunodeficiency that increase infection probability), and surgical team experience (e.g., surgeon proficiency independent of nursing training). To control for these factors, we adjusted for these covariates using multivariable regression: surgical site infection rates were 2.3% (7/300 procedures) in the BOPPPS group compared to 5.8% (17/295 procedures) in the control group (Fisher’s exact test = 4.36, *p* = 0.037; adjusted OR = 0.38, 95% CI: 0.16–0.89, *p* = 0.026). Mean operative time was reduced by 12.4 min (95% CI: 8.2–16.6 min, *t* = 5.78, *p* < 0.001; adjusted β = −11.9, 95% CI: −16.1 to −7.7, *p* < 0.001) for procedures involving BOPPPS-trained nurses.

No significant differences were observed in 30-day readmission rates between groups: 3.1% (9/290 patients) in the BOPPPS group vs. 3.5% (10/286 patients) in the control group (χ^2^ = 0.12, *p* = 0.729, RR = 0.89, 95% CI: 0.39–2.03). This non-significant trend may be attributed to factors unrelated to nursing training (e.g., post-discharge patient compliance, ward nursing care), and requires longer follow-up to verify potential long-term effects.

## 4 Discussion

This study provides compelling evidence that implementation of a BOPPPS-based teaching model in operating room nursing education yields substantial improvements in clinical competencies, team performance, and patient safety outcomes. The observed effect sizes across multiple domains exceed those reported in previous educational intervention studies, suggesting that the structured, learner-centered approach of BOPPPS is particularly well-suited to the complex learning environment of surgical settings (21).

The magnitude of improvement in OSCE scores (Cohen’s *d* = 1.32) represents a large effect size that translates into meaningful clinical benefits. Particularly noteworthy is the domain-specific analysis revealing the greatest improvements in sterile technique maintenance and communication skills, both critical factors in preventing surgical complications. These findings align with recent research by Al Abbas et al. demonstrating that enhanced team performance on surgical safety protocols correlates with reduced mortality and morbidity (22). The accelerated learning curve observed in the BOPPPS group, achieving competency milestones approximately 4 weeks earlier than controls, has important implications for workforce development and training efficiency in healthcare systems facing nursing shortages.

The 54% reduction in overall operational error rates among BOPPPS-trained nurses represents a clinically significant improvement in patient safety. This finding is particularly relevant given evidence that preventable errors in surgical settings often stem from cognitive and teamwork failures rather than technical skill deficits (19). The specific reduction in sterile technique breaches (60% lower than controls) directly addresses a major contributor to surgical site infections, which affect 2–5% of surgical patients and significantly increase healthcare costs and patient morbidity. Our results suggest that the systematic approach of BOPPPS, with its emphasis on pre-assessment, participatory learning, and immediate feedback, creates more robust mental models for error prevention compared to traditional observational learning.

The superiority of the BOPPPS model in preparing nurses for high-pressure surgical environments can be attributed to several key mechanisms. First, the bridge-in phase using authentic surgical videos appears to enhance situational awareness and pattern recognition, crucial skills for anticipating complications and surgeon needs. This finding corroborates research by Brogaard et al. showing that video-based learning improves clinical decision-making in acute care settings (20). Second, the structured pre-assessment component identifies individual learning needs, enabling targeted skill development rather than the one-size-fits-all approach of traditional teaching.

The participatory learning phase’s emphasis on graduated complexity and stress inoculation appears particularly beneficial for developing resilience and maintaining performance under pressure. Our gradient design—from static fundamental skills (Week 1) to high-pressure interprofessional simulation (Week 3)—aligns with the “deliberate practice” framework, which posits that expertise develops through progressive challenge rather than repetitive simple tasks (10). For example, Week 3’s emergency scenarios (e.g., hemorrhagic shock) forced students to apply technical skills under stress, an experience that traditional lecture-based teaching cannot replicate. This may explain why BOPPPS-trained nurses showed faster emergency response times (as seen in OSCE emergency station scores) and better stress resilience in clinical practice. Studies in high-reliability industries have long recognized that expertise develops through deliberate practice with progressively challenging scenarios (10). Our findings suggest similar principles apply to surgical nursing education. The integration of interprofessional simulation scenarios mirrors the actual OR environment more closely than traditional skills lab practice, potentially explaining the improved team integration scores reported by surgeons.

Compared to traditional teaching methods that rely heavily on passive observation and variable clinical exposure, BOPPPS provides consistent, comprehensive learning experiences. The model’s systematic approach addresses the “see one, do one, teach one” paradigm’s well-documented limitations, including inconsistent quality of instruction, inadequate practice opportunities, and lack of objective competency assessment (23). Furthermore, the immediate post-assessment and structured feedback components of BOPPPS align with contemporary understanding of how adults learn complex psychomotor and cognitive skills most effectively.

The versatility of the BOPPPS framework was demonstrated through its successful application across diverse surgical contexts. In minimally invasive procedures, the model proved particularly effective in developing spatial reasoning and hand-eye coordination skills necessary for laparoscopic assistance. Students trained with BOPPPS showed 40% faster adaptation to two-dimensional visualization and instrument manipulation compared to controls, consistent with research showing structured training accelerates laparoscopic skill acquisition (24).

Emergency surgical scenarios presented unique challenges that the BOPPPS model addressed through targeted design: pre-assessment identified gaps in emergency protocol knowledge (e.g., 40% of students missed key steps in hemorrhage control), allowing Week 3’s simulation to focus on these deficits. The addition of pressure factors (e.g., time constraints, equipment failure) in emergency training built “stress immunity”—a skill critical in ORs where delays in emergency response increase patient risk. Post-assessment data confirmed this: BOPPPS-trained nurses responded 35% faster to emergency situations and made fewer critical errors in protocol execution, aligning with evidence that simulation-based team training enhances crisis resource management skills (25).

Orthopedic procedures requiring specialized positioning and equipment handling also benefited from the BOPPPS approach. The model’s emphasis on hands-on practice with actual orthopedic instrumentation and positioning devices resulted in significant reductions in setup time and equipment-related delays. Similarly, in cardiac surgery contexts where perfusion management and sophisticated monitoring are critical, BOPPPS-trained nurses demonstrated superior ability to anticipate and respond to hemodynamic changes, potentially contributing to the observed reduction in operative times.

The mechanisms through which BOPPPS training translates to improved patient safety outcomes appear multifaceted. Enhanced communication skills, as evidenced by higher surgeon satisfaction ratings and reduced communication-related errors, likely play a central role. Research by Lingard et al. identified communication failures as contributing factors in 30% of surgical adverse events (26). The BOPPPS model’s emphasis on structured communication practice and role clarity directly addresses these vulnerabilities.

The discrepancy between objective communication error rates (Table 3: no significant difference) and subjective surgeon satisfaction (Table 4) (significant advantage) merits discussion. This may reflect differences in communication “quality” vs. “quantity”: BOPPPS training focused on proactive, standardized communication [e.g., using SBAR (Situation-Background-Assessment-Recommendation) format], which improved surgeon experience even if the total number of errors (e.g., delayed updates) did not decrease significantly. This aligns with surgical team research showing that “effective communication” is defined more by relevance and timeliness than error absence (26).

The observed reduction in surgical site infections can be attributed to multiple factors including improved adherence to sterile technique, better team coordination during critical moments, and enhanced situational awareness preventing contamination events. The participatory learning component’s focus on infection control practices under realistic conditions appears to create more durable behavior change compared to didactic instruction alone. This finding is supported by recent systematic reviews showing simulation-based infection control training significantly improves adherence to prevention protocols (25).

Team collaboration improvements likely stem from the model’s interprofessional learning components and emphasis on psychological safety during training. The structured debriefing sessions using the PEARLS framework appear to foster a culture of continuous improvement and open communication that persists into clinical practice. This aligns with research by Arad et al. demonstrating that psychological safety in OR teams correlates with better patient outcomes and staff satisfaction (27). The flattening of traditional hierarchies during BOPPPS training sessions may contribute to more effective speaking-up behaviors when patient safety concerns arise.

Our findings build upon and extend previous research on BOPPPS applications in healthcare education while introducing several innovative elements. Unlike previous studies that primarily measured knowledge acquisition and self-reported confidence, our research provides objective evidence of clinical performance improvements and patient outcomes. The effect sizes observed exceed those reported in recent meta-analyses of simulation-based surgical education (pooled effect size 0.71) and team training interventions (pooled effect size 0.58), suggesting synergistic benefits of the comprehensive BOPPPS approach (28).

The integration of real-time performance tracking and systematic error analysis represents a methodological advance over previous BOPPPS studies. While earlier research relied heavily on end-of-training assessments, our continuous monitoring approach captured the learning trajectory and identified critical transition points where additional support prevented skill decay. This finding has important implications for designing booster training and competency maintenance programs.

Our study’s focus on patient-centered outcomes addresses a critical gap in educational research. While Wang et al. demonstrated BOPPPS effectiveness for newly recruited nurses’ core competencies, they did not examine impacts on patient safety metrics (29). Similarly, although Ma et al. showed BOPPPS improved surgical nursing knowledge and critical thinking, clinical outcome data were lacking (30). By demonstrating reduced infection rates and operative times, our research provides the outcomes-based evidence necessary for institutional adoption and resource allocation decisions.

Several limitations warrant consideration when interpreting our findings. First, the single-center design may limit generalizability to institutions with different resources, patient populations, or surgical volumes. Multi-site replication studies are needed to validate the BOPPPS model’s effectiveness across diverse healthcare settings. Second, the 6-month follow-up period, while sufficient to demonstrate initial outcomes, cannot address long-term skill retention or career development impacts. Longitudinal studies tracking participants through their first years of independent practice would provide valuable insights into the durability of BOPPPS training effects.

The quasi-experimental design with non-equivalent control groups, while pragmatic given the constraints of clinical education scheduling, introduces potential selection bias. The use of convenience sampling based on clinical rotation schedules rather than random assignment represents a significant limitation. Students in different rotation periods might have different levels of motivation, prior informal exposure to surgical environments, or seasonal variations in clinical experiences. We attempted to minimize these effects by ensuring both groups received training during similar academic periods and by controlling for baseline characteristics in our analyses. Future research employing cluster randomization at the institutional level could address these concerns. Although baseline characteristics were well-balanced, the lack of randomization means that unmeasured confounders related to rotation timing or student self-selection might influence outcomes. Although baseline characteristics were well-balanced, unmeasured confounders such as motivation levels or learning style preferences might influence outcomes. Future research employing cluster randomization at the institutional level could address these concerns. Additionally, the Hawthorne effect may have influenced performance in both groups, though this would likely bias results toward the null hypothesis. Resource requirements for BOPPPS implementation represent a practical limitation. The model requires substantial faculty development, simulation facilities, and protected education time that may challenge resource-constrained institutions. Cost-effectiveness analyses comparing BOPPPS implementation costs against prevented complications and efficiency gains would inform adoption decisions. Future research should also explore technology-enhanced delivery methods, such as virtual reality simulation and remote coaching, to improve scalability and accessibility. Finally, our study focused on nursing education, but the surgical team’s interdisciplinary nature suggests potential benefits from interprofessional BOPPPS applications. Future research should examine outcomes when surgeons, anesthesiologists, and nurses train together using the BOPPPS framework, potentially yielding even greater improvements in team performance and patient safety.

## 5 Conclusion

This study demonstrates that implementation of a BOPPPS-based clinical teaching model in operating room nursing education produces substantial improvements in clinical competencies, surgical team performance, and patient safety outcomes. The structured, learner-centered approach of BOPPPS addresses critical gaps in traditional surgical nursing education, resulting in faster skill acquisition, reduced error rates, enhanced team collaboration, and measurable benefits for patient care. These findings provide robust evidence supporting the adoption of BOPPPS methodology as a standard framework for OR nursing education, with potential applications extending to broader surgical team training initiatives. Healthcare institutions seeking to enhance surgical safety and efficiency should consider investing in BOPPPS implementation as an evidence-based strategy for workforce development and quality improvement.

## Data Availability

The raw data supporting the conclusions of this article will be made available by the authors, without undue reservation.

## References

[1] Gillespie B Harbeck E Kang E Steel C Fairweather N Chaboyer W. Correlates of non-technical skills in surgery: a prospective study. *BMJ Open.* (2017) 7:e014480. 10.1136/bmjopen-2016-014480 28137931 PMC5293872

[2] Mitchell L Flin R Yule S Mitchell J Coutts K Youngson G. Thinking ahead of the surgeon. An interview study to identify scrub nurses’ non-technical skills. *Int J Nurs Stud.* (2011) 48:818–28. 10.1016/j.ijnurstu.2010.11.005 21190685

[3] Sørensen J Østergaard D LeBlanc V Ottesen B Konge L Dieckmann P Design of simulation-based medical education and advantages and disadvantages of in situ simulation versus off-site simulation. *BMC Med Educ.* (2017) 17:20. 10.1186/s12909-016-0838-3 28109296 PMC5251301

[4] Tan S Marlow N Field J Altree M Babidge W Hewett P A randomized crossover trial examining low- versus high-fidelity simulation in basic laparoscopic skills training. *Surg Endosc.* (2012) 26:3207–14. 10.1007/s00464-012-2326-0 22648100

[5] Alsadoun L Sanipini S Khleif R Ashfaq A Shehryar A Berhane KA Evaluating the impact of the World Health Organization’s surgical safety checklist on clinical outcomes and implementation strategies: a systematic review. *Cureus*. (2024) 16:e69875. 10.7759/cureus.69875 39435236 PMC11493453

[6] Davenport D Henderson W Mosca C Khuri S Mentzer R. Risk-adjusted morbidity in teaching hospitals correlates with reported levels of communication and collaboration on surgical teams but not with scale measures of teamwork climate, safety climate, or working conditions. *J Am Coll Surg.* (2007) 205:778–84. 10.1016/j.jamcollsurg.2007.07.039 18035261

[7] Ma X Zeng D Wang J Xu K Li L. Effectiveness of bridge-in, objective, pre-assessment, participatory learning, post-assessment, and summary teaching strategy in Chinese medical education: a systematic review and meta-analysis. *Front Med.* (2022) 9:975229. 10.3389/fmed.2022.975229 36186766 PMC9521335

[8] Liu X Lu C Zhu H Wang X Jia S Zhang Y Assessment of the effectiveness of BOPPPS-based hybrid teaching model in physiology education. *BMC Med Educ.* (2022) 22:217. 10.1186/s12909-022-03269-y 35354465 PMC8966603

[9] Zhu J Xiao H Zhou R Gan X Gou Q Tie H. The efficacy of the BOPPPS teaching model in clinical and health education: a systematic review and meta-analysis. *BMC Med Educ.* (2025) 25:997. 10.1186/s12909-025-07274-9 40604993 PMC12224838

[10] Li Z Cai X Zhou K Qin J Zhang J Yang Q Effects of BOPPPS combined with TBL in surgical nursing for nursing undergraduates: a mixed-method study. *BMC Nurs.* (2023) 22:112. 10.1186/s12912-023-01281-1 37088853 PMC10122814

[11] Yoshikawa A Ohtsuka H Aoki K Tashiro N Togo S Komaba K Simulation-based infection prevention and control training for medical and healthcare students: a systematic review. *Front Med.* (2025) 12:1529557. 10.3389/fmed.2025.1529557 40438369 PMC12116496

[12] Chen C Shi X Yin S Fan N Zhang T Zhang X Application of the online teaching model based on BOPPPS virtual simulation platform in preventive medicine undergraduate experiment. *BMC Med Educ.* (2024) 24:1255. 10.1186/s12909-024-06175-7 39501207 PMC11536896

[13] Schmutz J Manser T. Do team processes really have an effect on clinical performance? A systematic literature review. *Br J Anaesth.* (2013) 110:529–44. 10.1093/bja/aes513 23454826

[14] Wei L Yu X Wang Y Shan N. Application of BOPPPS and flipped classroom joint teaching model into clinical practice ability of obstetrics and gynecology residents in standardized training. *BMC Med Educ.* (2025) 25:655. 10.1186/s12909-025-07246-z 40329285 PMC12057154

[15] Paige J Garbee D Bonanno L Kerdolff K. Qualitative analysis of effective teamwork in the operating room (OR). *J Surg Educ.* (2021) 78:967–79. 10.1016/j.jsurg.2020.09.019 33160940

[16] Etherington C Wu M Cheng-Boivin O Larrigan S Boet S. Interprofessional communication in the operating room: a narrative review to advance research and practice. *Can J Anaesth.* (2019) 66:1251–60. 10.1007/s12630-019-01413-9 31140044

[17] Al Abbas A Sankaranarayanan G Polanco P Cadeddu J Daniel W Palter V The operating room black box: understanding adherence to surgical checklists. *Ann Surg.* (2022) 276:995–1001. 10.1097/SLA.0000000000005695 36120866

[18] Catchpole K Mishra A Handa A McCulloch P. Teamwork and error in the operating room: analysis of skills and roles. *Ann Surg.* (2008) 247:699–706. 10.1097/SLA.0b013e3181642ec8 18362635

[19] Aggarwal R Moorthy K Darzi A. Laparoscopic skills training and assessment. *Br J Surg.* (2004) 91:1549–58. 10.1002/bjs.4816 15547882

[20] Brogaard L Kierkegaard O Hvidman L Jensen K Musaeus P Uldbjerg N The importance of non-technical performance for teams managing postpartum haemorrhage: video review of 99 obstetric teams. *BJOG.* (2019) 126:1015–23. 10.1111/1471-0528.15655 30771263

[21] McGaghie W Issenberg S Cohen E Barsuk J Wayne D. Does simulation-based medical education with deliberate practice yield better results than traditional clinical education? A meta-analytic comparative review of the evidence. *Acad Med.* (2011) 86:706–11. 10.1097/ACM.0b013e318217e119 21512370 PMC3102783

[22] Stone R Cooke M Mitchell M. Undergraduate nursing students’ use of video technology in developing confidence in clinical skills for practice: a systematic integrative literature review. *Nurse Educ Today.* (2020) 84:104230. 10.1016/j.nedt.2019.104230 31689584

[23] Kotsis S Chung K. Application of the see one, do one, teach one concept in surgical training. *Plast Reconstr Surg.* (2013) 131:1194–201. 10.1097/PRS.0b013e318287a0b3 23629100 PMC4785880

[24] Wiegmann D ElBardissi A Dearani J Daly R Sundt T. Disruptions in surgical flow and their relationship to surgical errors: an exploratory investigation. *Surgery.* (2007) 142:658–65. 10.1016/j.surg.2007.07.034 17981185

[25] Lee S Yang I. Scenario-based simulation training as a strategy to improve infection prevention and control adherence: a quasi-experimental study. *Appl Nurs Res.* (2025) 83:151966. 10.1016/j.apnr.2025.151966 40473368

[26] Lingard L Espin S Whyte S Regehr G Baker G Reznick R Communication failures in the operating room: an observational classification of recurrent types and effects. *Qual Saf Health Care.* (2004) 13:330–4. 10.1136/qhc.13.5.330 15465935 PMC1743897

[27] Arad D Finkelstein A Rozenblum R Magnezi R. Patient safety and staff psychological safety: a mixed methods study on aspects of teamwork in the operating room. *Front Public Health.* (2022) 10:1060473. 10.3389/fpubh.2022.1060473 36620282 PMC9816421

[28] Cook D Hatala R Brydges R Zendejas B Szostek J Wang A Technology-enhanced simulation for health professions education: a systematic review and meta-analysis. *JAMA.* (2011) 306:978–88. 10.1001/jama.2011.1234 21900138

[29] Wang Y Chen Y Wang L Wang W Kong X Li X. Assessment of the effectiveness of the BOPPPS model combined with case-based learning on nursing residency education for newly recruited nurses in China: a mixed methods study. *BMC Med Educ.* (2024) 24:215. 10.1186/s12909-024-05202-x 38429761 PMC10908075

[30] Ma B Luo X Shen X. Evaluation on effectiveness of BOPPPS method in evidence-based medicine teaching. *Chin J Evidence-Based Med.* (2019) 19: 904–8. 10.7507/1672-2531.201901061 38268090

